# Therapeutic potential of Coptis chinensis for arthritis with underlying mechanisms

**DOI:** 10.3389/fphar.2023.1243820

**Published:** 2023-08-11

**Authors:** Mengyuan Li, Fei Tian, Jinling Guo, Xiankuan Li, Lin Ma, Miaomiao Jiang, Jing Zhao

**Affiliations:** ^1^ Haihe Laboratory of Modern Chinese Medicine, Tianjin, China; ^2^ National Key Laboratory of Chinese Medicine Modernization, Tianjin University of Traditional Chinese Medicine, Tianjin, China; ^3^ School of Chinese Materia Medica, Tianjin University of Traditional Chinese Medicine, Tianjin, China; ^4^ Department of Geriatric, Fourth Teaching Hospital of Tianjin University of Traditional Chinese Medicine, Tianjin, China

**Keywords:** Coptis chinensis, arthritis, berberine, Wnt1/β-catenin signaling pathway, PI3K/Akt/mTOR signaling pathway

## Abstract

Arthritis is a common degenerative disease of joints, which has become a public health problem affecting human health, but its pathogenesis is complex and cannot be eradicated. Coptis chinensis (CC) has a variety of active ingredients, is a natural antibacterial and anti-inflammatory drug. In which, berberine is its main effective ingredient, and has good therapeutic effects on rheumatoid arthritis (RA), osteoarthritis (OA), gouty arthritis (GA). RA, OA and GA are the three most common types of arthritis, but the relevant pathogenesis is not clear. Therefore, molecular mechanism and prevention and treatment of arthritis are the key issues to be paid attention to in clinical practice. In general, berberine, palmatine, coptisine, jatrorrhizine, magnoflorine and jatrorrhizine hydrochloride in CC play the role in treating arthritis by regulating Wnt1/β-catenin and PI3K/AKT/mTOR signaling pathways. In this review, active ingredients, targets and mechanism of CC in the treatment of arthritis were expounded, and we have further explained the potential role of AHR, CAV1, CRP, CXCL2, IRF1, SPP1, and IL-17 signaling pathway in the treatment of arthritis, and to provide a new idea for the clinical treatment of arthritis by CC.

## Graphical Abstract

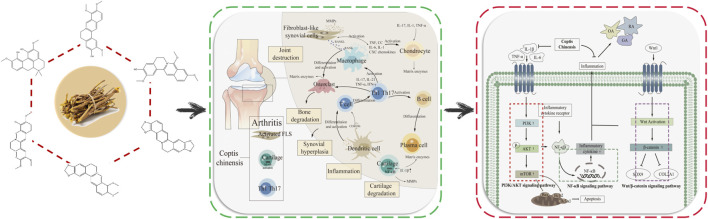

## 1 Introduction

Arthritis is a series of inflammatory diseases occurring in human joints or surrounding tissues, and it can lead to joint disability in serious cases. The incidence of arthritis is increasing year by year. There are more than 355 million arthritis patients worldwide, and the number is still increasing (Jia, 2021). Rheumatoid arthritis (RA) and osteoarthritis (OA) have the highest incidence (Wang, 2020; She, 2020), and the pathogenesis of them is complex. RA is a chronic autoimmune disease caused by synovial joint inflammation, which gradually leads to joint damage, cartilage degradation, disability, with a high disability rate in the later stage of the disease (Bird et al., 2022). OA is the most common type of arthritis associated with age and occurs most often in the elderly (Roškar and Hafner-Bratkovič, 2022). The causes of RA, gouty arthritis (GA) and OA are varied, mainly caused by the combined effects of congenital genetic factors and acquired environmental factors, and the related molecular mechanisms are complicated. The prevention and treatment of these three types of arthritis are the focus of research. Currently, arthritis cannot be cured clinically, and joint function can only be maintained through drug therapy. However, long-term treatment with single drug or combined immunosuppressive drugs have great limitations and cause adverse reactions. Therefore, it has important significance to explore the pathogenesis of arthritis and develop natural drugs to treat arthritis.

Coptis chinensis (CC) is the dried rhizome of the Ranunculaceae plant Coptis chinensis Franch., Coptis deltoidea C.Y.Cheng et Hsiao, or Coptis teeta Wall (National Pharmacopoeia Commission, 2020), which has antibacterial, anti-inflammatory, antioxidant, anti-tumor, antiarrhythmic and other pharmacological effects (Gai et al., 2018). CC is commonly used in clinical treatment of cardiovascular and cerebrovascular diseases, diabetes, cancer and other diseases (Fu et al., 2021). CC contains more than 130 chemical components, mainly including alkaloids, coumarins, organic acids, and flavonoids (Zhou et al., 2020). CC has good therapeutic effect on RA, OA, GA (Yue et al., 2019; Huang et al., 2021; Zhang et al., 2022; Elkomy et al., 2022). The alkaloid berberine, palmatine, coptisine, jatrorrhizine, magnoflorine and jatrorrhizine hydrochloride show significant antibacterial and anti-inflammatory effects. Studies have shown that berberine can effectively treat RA, OA and GA, mainly by reducing the level of inflammatory factors, regulating intestinal flora, promoting uric acid excretion, and improving the inflammatory response damage of joints and their surrounding tissues (Fan et al., 2021; Xu and Li, 2021). At the molecular level, CC can improve arthritis by regulating Wnt1/β-catenin, PI3K/AKT/mTOR and NF-κB signaling pathways, inhibiting the expression of pro-inflammatory factors such as interleukin-1β (IL-1β), interleukin-6 (IL-6), tumor necrosis factor-α (TNF-α), inducible nitric oxide synthase (iNOS), etc (Zhao et al., 2014; Zhou et al., 2019; Shen et al., 2020; Chen et al., 2023). Berberine, palmatine, coptisine and other components are the main components of CC in the treatment of arthritis. Interleukin-10 (IL-10), IL-1β, mitogen-activated protein kinase (MAPK), IL-6, matrix metalloproteinase-3 (MMP-3), TNF-α and other targets have been confirmed to play important roles in the treatment of arthritis by CC. The regulation of IL-17 signaling pathway in chondrocytes could inhibit the overexpression and activation of key proteins, such as IL-17RA, ACT1 and TRAF6, which could improve the occurrence of cartilage inflammation in OA. Research has shown that aryl hydrocarbon receptor (AHR), caveolin-1 (CAV1), c-reactive protein (CRP), C-X-C motif chemokine 2 (CXCL2), interferon regulatory factor-1 (IRF1), secreted phosphoprotein 1 (SPP1) and other targets are key targets in the pathogenesis of arthritis (Chen et al., 2022; Wang et al., 2022; Sakthiswary et al., 2022; Zhu et al., 2022; Li et al., 2023; Yang et al., 2023), but role of them in the treatment of arthritis by CC has not been verified and further clarification is needed.

Studies have shown that chemical drugs used in the treatment of arthritis have different degrees of toxicity and side effects. CC, as a natural Chinese herbal medicine with low toxicity of antibacterial and anti-inflammatory, is feasible to develop as a drug for treating arthritis. However, there are few studies on the treatment of arthritis by CC. Therefore, it is of great significance to clarify the mechanism of CC in the treatment of arthritis, which can provide new research directions for clinical drug development. We have elaborated the active ingredients, targets and mechanism in the treatment of RA, OA and GA by CC, revealed the potential targets and related pathways of CC in the treatment of arthritis, and provided new insights into the study of the molecular mechanism of CC in treating arthritis. In this review, the chemical components, targets and pathways of CC in the treatment of arthritis were discussed in detail, the molecular mechanism of CC in treating arthritis was elaborated, and the potential therapeutic targets were analyzed, providing new ideas for clinical prevention and treatment of arthritis.

## 2 The pathogenesis of three types of arthritis

**FIGURE 1:**
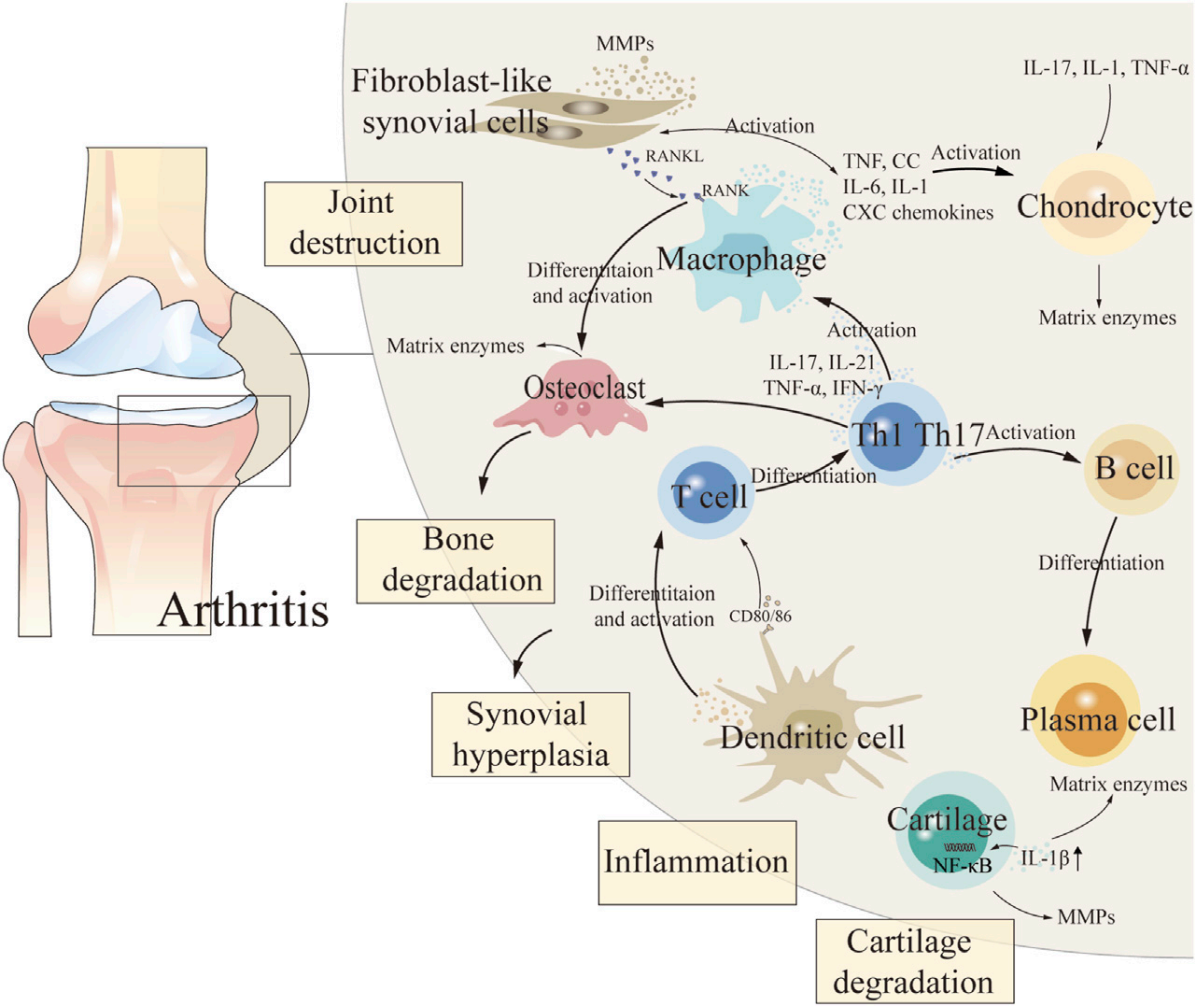
The pathogenesis of arthritis.

The etiology of arthritis is complex and relevant pathogenesis has not been clarified (Chen et al., 2021). During the pathogenesis of arthritis, fibroblast-like synovial cells (FLS), chondrocytes, intrinsic immune cells (dendritic cells and macrophages), and adaptive immune cells (T and B cells) in synovial tissue release a variety of cytokines that lead to persistent destruction of cartilage and subchondral bone, thereby exacerbating the degree of arthritis (So and Martinon, 2017; Xing, 2021; Xu, 2022) (Figure 1 demonstrated the pathogenesis of arthritis). Non-steroidal anti-inflammatory drugs, anti-rheumatism drugs, traditional Chinese medicine (TCM) compounds and other drugs are commonly used in clinical treatment of arthritis. But chemical therapy cannot cure arthritis, and it can only relieve joint function, and long-term use of these treatments can cause relatively significant toxic and side effects, causing liver, kidney and cardiovascular toxicity (Li, 2022; Yu, 2022). TCM has unique advantages which due to its characteristics of multiple components, low toxicity, few side effects and good curative effects in the treatment of RA and OA (Li et al., 2022; Meng et al., 2022). The effective components of TCM show great potential in the treatment of arthritis by inhibiting inflammatory response, alleviating oxidative stress, regulating chondrocyte metabolism and regulating related signaling pathways (Li et al., 2017).

### 2.1 Rheumatoid arthritis

RA is an autoimmune inflammatory disease with systemic sequelae (Hyndman, 2017), the main symptoms of which are synovial inflammation, production of rheumatoid factors and antibodies against citrulline proteins, and destruction of cartilage and bone (McInnes and Schett, 2011). The pathogenesis of RA is confused and difficult curative ratio, which is mainly caused by excessive proliferation of synovial cells, increased levels of inflammatory factors and abnormal toll-like receptor signaling pathway (Wen, 2022). Inflammatory response is an important pathological process of RA. Abnormal secretion of proinflammatory cytokines, chemokines and proteases will disturb the balance of the body and lead to cartilage and bone damage (Fang et al., 2020). The pathological feature of RA is the infiltration of synovial inflammatory cells in multiple joints. Nuclear factor кB receptor activating factor (RANKL), prostaglandins and matrix metalloproteinases (MMPs) are induced by pro-inflammatory cytokines, including TNF-α, IL-6 and interleukin-1 (IL-1), causing joint pain and swelling (Wallach, 2016). At the molecular level, MAPK, TLR7/NF-кB and apoptosis signaling pathways are the main signaling pathways involved in regulating the invasion and abnormal behavior of RA-FLS cells (Zheng, 2012; Bottini and Firestein, 2013). Currently, the main treatment is to reduce inflammation and relieve pain (Fan et al., 2020). Modern studies of TCM have shown that a variety of monomer components of TCM have the efficacy of treating RA (Yao et al., 2023), including sinomenine, artemisinin, total glucosides of paeony and berberine (Ou et al., 2010; Wang et al., 2011; Xia and Li, 2017; Sharma et al., 2022).

### 2.2 Osteoarthritis

OA is the most common type of arthritis that causes joint pain and disability, and it has a high incidence (Li et al., 2022). The main characteristics of OA are cartilage degeneration, synovial hyperplasia, osteophyte formation and subchondral osteosclerosis, but the pathogenesis has not been clearly defined (Chen et al., 2022). The occurrence and development of OA is closely related to degradation of matrix and release of bioactive substances, which promote the release of MMPs and eventually lead to chondrolysis (Uthman et al., 2003). Inflammatory factors and receptors are involved in the occurrence and development of OA, which can cause degenerative changes of chondrocytes through MAPK/ERK, JAK2/STAT3, NF-κB, Wnt/β-catenin and PI3K/AKT signaling pathways (Hwang et al., 2005; Roemer et al., 2011; Min et al., 2017). Chondrocytes are the source and target of pro-inflammatory cytokines in OA. Pro-inflammatory cytokine interleukin-1 is an important inflammatory mediator secreted by early OA and a key inflammatory cytokine involved in the pathogenesis of OA (Li et al., 2019; Yang et al., 2021). IL-1β mainly affects the metabolism of articular cartilage extracellular matrix and chondrocytes, and plays an important role in the pathogenesis of OA by inducing excessive release of inflammatory mediators cycloooxygenase-2 (COX-2) and iNOS, and overexpression of cartilage MMPs. IL-1β, TNF-α and IL-6 are three highly expressed inflammatory cytokines in OA joints, which are actively produced by chondrocytes, synovial cells, macrophages and osteoblasts, and can be used as indicators of the progression of OA (Zhou et al., 2016a).

### 2.3 Gouty arthritis

GA is an inflammatory reactive disease that causes joint pain due to the dysfunction of purine metabolism and uric acid (UA) excretion in the body (AbdullGaffar et al., 2020). The pathogenesis of GA is related to the inflammatory response caused by the deposition of monosodium urate (MSU) around the joint, which stimulates the synovial membrane to produce pathological reactions such as synovial vasodilation and leukocyte exudation, which mainly involve the mediation of MAPK and NF-κB signaling pathways and the activation of TNF-α, IL-1 and other inflammatory cytokines (Choe et al., 2014; Terkeltaub, 2017; Lv, 2020; Liu et al., 2022).

## 3 Chemical components and mechanism of Coptis chinensis in the treatment of arthritis

### 3.1 The chemical components of Coptis chinensis in treating arthritis

CC has obvious inhibitory effect on acute and chronic inflammatory reactions (Park et al., 2018). CC contains a variety of anti-inflammatory active ingredients, such as berberine, palmatine, coptisine, *etc.* (Hu et al., 2013; Wang et al., 2014; Zhou et al., 2015; Zhou et al., 2016; Zhou et al., 2017; Qiu et al., 2018; Sujitha et al., 2018; Wang et al., 2019; Wu et al., 2019; Zhou et al., 2019; Zhou et al., 2020; Shen et al., 2020; Yu et al., 2020; Fan et al., 2021; Cheng et al., 2022; Asila et al., 2022; Li et al., 2022; Kou et al., 2022) (Table 1), which can achieve anti-inflammatory effects mainly by inhibiting the activity of key proteins in the inflammatory signaling pathway and blocking the transmission of inflammatory signals (Hu and Mo, 2017; Geng, 2018). Berberine has a significant anti-inflammatory activity and can treat a variety of arthritis, especially RA and OA (Hu et al., 2010; Zhou et al., 2016c).

**TABLE 1:** Main chemical components of CC in treating arthritis and related pharmacological data.

Compounds	Classifications	Pathways/Targets	Animal/Cell	Effective dose	References
**Berberine**	OA	Caspase-3, ADAMTS5, MMP-13	Chondrocytes	10 μg/mL	Zhou et al. (2017a)
		TLR4/NF-κB signaling	SD rats/Primary articular chondrocytes	200 μM	Zhou et al. (2020b)
				75 μM	
		AMPK signaling	AMPKα1 global knockout (KO) mice/Chondrocytes	1.1 mg/mL	Li et al. (2022b)
				5, 25 μM	
		NF-κB pathway	SD rats/RAW 264.7 RCCs	120 μM	Kou et al. (2022)
				40 μM	
		p38/MAPK	SD rats/Primary articular chondrocytes	200 μM	Zhou et al. (2015)
				75 μM	
	RA	PI3K/AKT, Wnt1/β-catenin, AMPK/lipogenesis	SD rats	200 mg/kg	Shen et al. (2020)
		p38/ERK MAPK pathway	Primary FLS-RA	12.5, 25 μM	Wang et al. (2019a)
		ASK1/p38 signaling	RAW 264.7	25, 50, 75 µM	Sujitha et al. (2018)
			AA-SM cells		
	Septic arthritis	NF-κB/JNK-RANKL axis	Adult male mice	50, 100, 200 mg/kg	Asila et al. (2022)
	Adjuvant-induced arthritis (AIA)	AMPK/HIF-1α pathway	SD rats/Peritoneal macrophages	80 mg/kg	Yu et al. (2020)
				10 μM	
		AMPK/NF-кB pathway	SD rats	80 mg/kg	Zhou et al. (2019)
	Type II collagen-induced arthritis	VEGF, p-JNK, p-p38	SD rats	200 mg/kg	Wang et al. (2014a)
	GA	COX-2, NALP3, TGF-β	Acute gouty arthritis (AGA) patients	0.4 g/time	Fan et al. (2021)
**Palmatine**	GA	NF-κB/NLRP3, Nrf2 pathways	KM male mice/THP-1 cells	100 mg/kg	Cheng et al. (2022a)
				80 μM	
	OA	Wnt/β-catenin, Hedgehog pathways	New Zealand rabbits/Primary chondrocytes	100 mg/L	Zhou et al. (2016b)
				10, 25, 50, 100 mg/L	
**Coptisine**	GA	Caspase-1	Male Kun Ming mice/RAW264.7	2.91, 5.79, 11.61 mg/kg	Wu et al. (2019)
				1, 10, 30 μM	
**Jatrorrhizine**	Collagen-induced arthritis (CIA)	Anti-CII, IgG1	SD rats	8 mg/kg	Hu et al. (2013)
**Magnoflorine**				8.7 mg/kg	
**Jatrorrhizine hydrochloride**	RA	MAPK, ERK, p38	SD rats	50 mg/kg	Qiu et al. (2018)

#### 3.1.1 Berberine

Berberine is the main active ingredient in CC that plays an anti-inflammatory and antibacterial role. It can effectively treat a variety of arthritis by down-regulating the production and expression of various inflammatory mediators and inhibiting the activation of inflammatory pathways (He et al., 2018). Berberine has a strong anti-rheumatoid effect and can slow the progression of RA by targeting mitochondrial oxidative phosphorylation (Fan et al., 2018; Elkomy et al., 2022) confirmed that berberine could effectively inhibit RA inflammation. By inhibiting autophagy of FLS cells, berberine induces RA-FLSs cycle arrest in G0/G1 phase, induces RA-FLSs cell death, inhibits the expression of vascular endothelial growth factor, regulates the level of anti-inflammatory factors, and achieves the purpose of treating RA (Wang et al., 2014; Huang et al., 2021). Wang (2011) verified that berberine induced apoptosis of RA-FLSs mainly through the mechanism of up-regulating the expression of pro-apoptotic protein apoptosis regulator BAX (Bax), inhibiting the expression of anti-apoptotic proteins apoptosis regulator Bcl-2 (Bcl-2) and Bcl-xl, and promoting the activation of caspase-3, caspase-9 and PARP. (Wang et al., 2017) found that berberine could reduce the expression level of interleukin-17 (IL-17) and IL-6, promote the expression of IL-10 and transforming growth factor-β (TGF-β) in serum, and improve the clinical symptoms of RA. Berberine can significantly suppress the activation of p-ERK, p-p38 and p-JNK, reduce the destruction of inflammatory cells on joint tissues, and exert anti-RA activity (Wang et al., 2014). During the treatment of RA, berberine reduced the expression levels of TNF-α, IL-17, interferon-γ (IFN-γ), MMPs and RAR-related orphan receptor γt (RORγt) (Sharma et al., 2022). Studies have shown that berberine treats RA by specifically inhibiting T cells, involving the balance between Treg and Th17 cells, providing a potential target for berberine in the treatment of arthritis (Li et al., 2017; Vita et al., 2021).

(Xie et al., 2018) found that berberine could increase the enzymatic antioxidant levels, such as SOD, glutathione peroxidase, catalase and glutathione-S-transferase in osteoporosis rats, which is helpful to prevent osteoporosis. (Huang et al., 2021) found that berberine promoted the proliferation and activity of IL-1β-induced inflammatory degenerative chondrocytes by inhibiting cell inflammatory response and activating TGF-β/Smad2/3 signaling pathway, and reduced the degree of OA development. Berberine is a potential therapeutic drug for OA. MMPs play a significant role in OA-induced articular cartilage damage (Dean et al., 1989; Hu et al., 2011) showed that berberine could inhibit the expression of matrix metalloproteinase-1 (MMP-1), MMP-3 and matrix metalloproteinase-13 (MMP-13) and effectively treat OA. Connective tissue growth factor (CCN2) is abundantly expressed response. Berberine inhibits CCN2 to produce IL-1β by down-regulating ROS-mediated NF-κB signaling pathway in fibroblast synovial cells, regulates cartilage damage and alleviates OA (Liu et al., 2015). Berberine can inhibit the expression of NO, prostaglandin E2 (PGE2), iNOS, COX-2, MMP-3 and MMP-13 induced by IL-1β, downregulate the expression of inflammatory mediators and reduce the inflammatory response in chondrocytes (Zhou et al., 2016).

By inhibiting the NOD-like receptor thermal protein domain 3 (NLRP3)/Toll-like receptor signaling pathway, berberine downregulated the expression levels of IL-2, IL-6 and TNF-α, and alleviated the degree of ankle swelling in GA mice (Jian et al., 2020). Berberine reduces the expression levels of NLRP3, TNF-α and IL-1β and the level of intracellular reactive oxygen species, thereby reducing MSU crystal-induced inflammation in rats (Liu et al., 2016). Berberine improves the acute symptoms of GA by inhibiting the activity of joint elastase and thereby inhibiting the infiltration of joint synovium neutrophils (Dinesh and Rasool, 2017). The increase of serum UA level is the key to AGA attack. Berberine can dilate blood vessels, improve blood flow and increase the expression of human urate transporter, thus increasing blood UA excretion and reducing UA level in the body. In addition, berberine can also improve insulin resistance and inhibit UA synthesis (Jin et al., 2019; Rondanelli et al., 2020). IL-1β is considered to be the initiating factor of AGA inflammatory response (Li et al., 2019), PGE2 has a strong inflammatory effect and is involved in the whole process of GA inflammatory response, and COX-2 is a key enzyme in the synthesis of PGE2 in the body (Liu et al., 2019). Fan et al. (Fan et al., 2021) showed that assisted treatment of AGA of berberine could significantly inhibit the expressions of inflammatory factors IL-1β, COX-2, nucleotide-binding oligomerization domain-like receptor 3 (NALP3) and TGF-β, reduce the levels of CRP, ESR and UA, and effectively relieve the symptoms of AGA.

#### 3.1.2 Palmatine

Palmatine has been proved to have antipyretic, antibacterial and anti-inflammatory activities, it has been used as an anti-inflammatory agent in clinical practice (Pathan et al., 2015; Zhou et al., 2017). Palmatine has a good effect in the treatment of OA, it can effectively inhibit the expression of MMP-1, MMP-3 and matrix metalloproteinase-9 (MMP-9) induced by IL-1β by blocking Wnt1/β-catenin and Hedgehog signaling pathways, and improve OA (Zhou, 2014). Palmatine can inhibit expression of IL-1β and MMPs, it promotes the expression of cyclopamine which is inhibitor of the Hedgehog signaling pathway and suppress Wnt/β-atenin signaling pathway, to exert protective effect on OA and possess potential antalgic effect (Zhou et al., 2016). Research has shown that palmatine can improve joint swelling and significantly inhibit the expression of IL-1β, IL-6, IL-18, TNF-α in joint tissue, block the infiltration of inflammatory cells into the synovium and joint cavity, to achieve the therapeutic effect of GA (Cheng et al., 2022).

#### 3.1.3 Other ingredients

Alkaloids in CC are the main anti-inflammatory active ingredients. Besides berberine and palmatine, coptisine, jarrorhizine hydrochloride and magnoflorine also play important roles in the anti-inflammatory effect of CC. The expression level of C-X-C motif chemokine 12 (CXCL12) in synovium of patients with OA was significantly increased, and C-X-C chemokine receptor 4 (CXCR4) was its receptor (Wei et al., 2010; Bragg et al., 2019). Coptisine, as a CXCR4 antagonist, inhibits the overexpression of ADAMTS4,5 in chondrocytes induced by CXCL12, improving cartilage degradation and subchondral bone damage (Yang et al., 2023). Coptisine inhibits the activation of NLRP3 inflammatory bodies by blocking caspase-1, which can be used to treat GA associated with NLRP3 inflammatory bodies (Wu et al., 2019). Jarrorhizine hydrochloride can significantly inhibit the expression levels of IL-1β, IL-6, IL-8, matrix metalloproteinase-2 (MMP-2), and MMP-3, suppress the proliferation, migration, and secretion of synovial cells, prevent bone destruction, and thus improve the severity of RA (Qiu et al., 2018). Magnoflorine has significant anti-inflammatory effect, which may improve RA by promoting the synthesis of proteoglycans in chondrocytes (Yue et al., 2013; Gui et al., 2015). Researches have shown that magnoflorine reduces IL-1β-induced inflammatory cytokine levels and inhibits inflammatory responses in AIA rats by regulating the PI3K/AKT/NF-κB signaling pathway (Shen et al., 2022). In a traumatic osteoarthritis model, magnoflorine can promote the proliferation, chondrogenesis and migration of cartilage progenitor cells by activating the chondrogenic signaling pathway, thereby directly reducing articular cartilage degeneration (Cai et al., 2020). By inhibiting the expression of TNF-α, IL-6, IL-1β, MCP-1, iNOS and IFN-β, magnoflorine improved the degree of joint destruction and macrophage infiltration in synovial tissue of CIA mice, and achieved the purpose of treating arthritis by inhibiting the activation of NF-κB and MAPK signaling pathways (Wang et al., 2023a).

### 3.2 The mechanism of Coptis chinensis in treating arthritis

**FIGURE 2:**
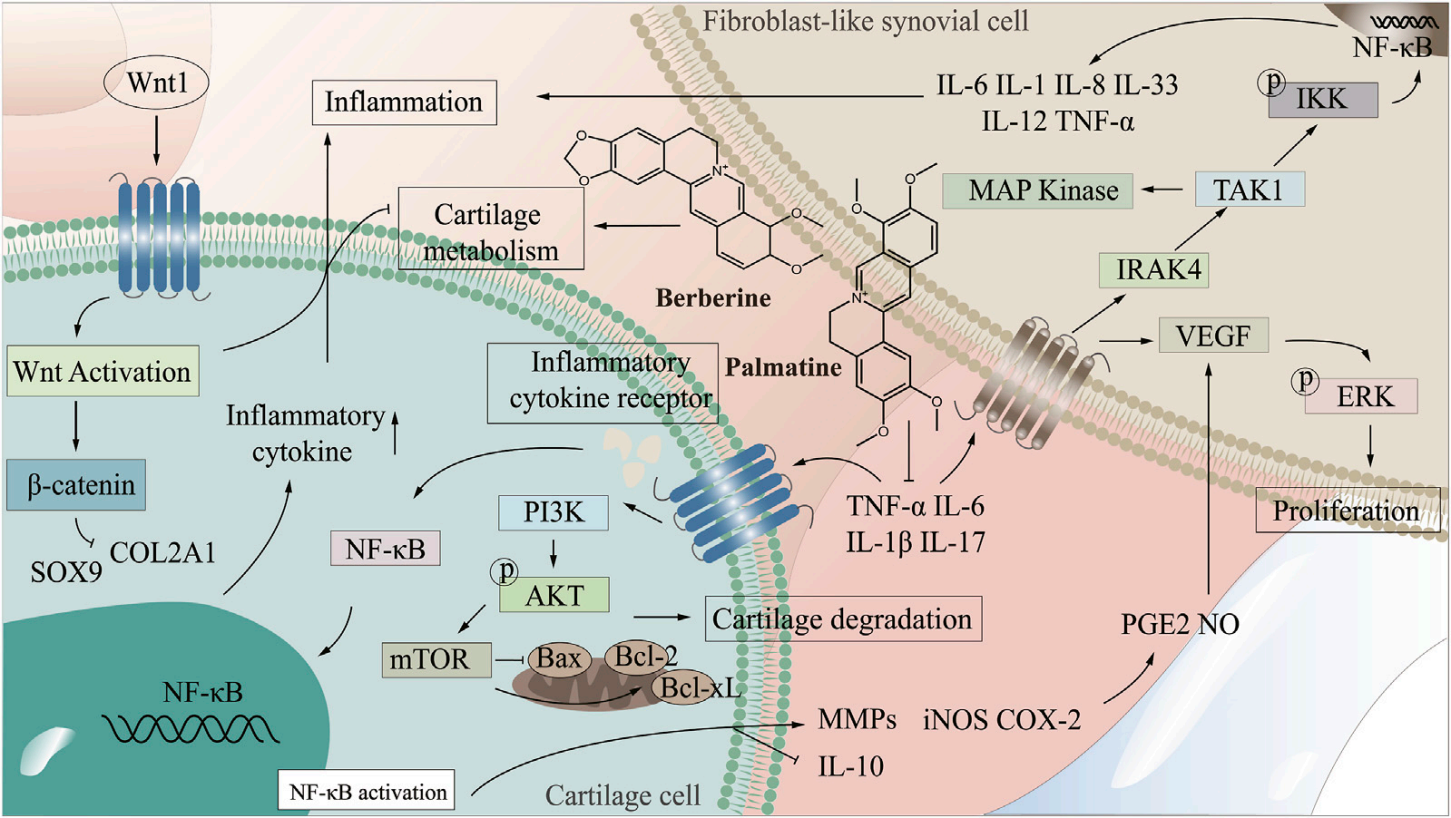
The mechanism of CC in the treatment of arthritis.

There are many uncomfortable symptoms in the clinical treatment of arthritis, and the development of natural drugs is greatly on demand. CC, as an antibacterial and anti-inflammatory Chinese medicine, presents an excellent potential for treating arthritis. By improving the targeting of CC to arthritic damaged tissues and enhancing the bioavailability of CC in treatment, it is promising to realize the development of novel natural medicines with enhanced curative effect and low side effects. Therefore, it is extremely important to clarify the mechanism of CC in treating arthritis. The NF-κB signaling pathway is one of the crucial pathways in the pathogenesis of arthritis, abnormal activation of which will lead to synovial inflammation, chondrocyte apoptosis and destruction (Qing et al., 2014; Wu, 2021; Zeng, 2022). PI3K/AKT/mTOR signaling pathway is a central regulator of cell growth, proliferation and cell cycle, and plays a significant role in chondrocyte degeneration (Xu et al., 2014). Wnt/β-catenin signaling pathway affects bone modeling and bone remodeling, especially the differentiation of osteoblasts, which may be a potential target for treating bone diseases (Wang et al., 2014). In the treatment of arthritis, CC can reduce the levels of IL-1β, TNF-α, IL-6, COX-2, NALP3 and TGF-β, regulate NF-κB and PI3K/AKT/mTOR signaling pathways, which promote the proliferation of articular chondrocytes, inhibit apoptosis, and enhance cell healing ability, thereby improving bone and joint, inhibiting bone destruction and reducing inflammation in joints and surrounding tissues (Fan et al., 2021; Jie et al., 2022; Liu et al., 2023), (Figure 2 demonstrated the mechanism of CC in the treatment of arthritis).

#### 3.2.1 Wnt1/β-catenin signaling pathway

Wnt1/β-catenin signaling pathway plays a key role in cell proliferation, differentiation and autoimmune regulation (Cici et al., 2019). Wnt1/β-catenin signaling pathway produces a significant role in tissue repair and joint homeostasis by regulating the activity of synovial cells, osteoblasts and chondrocytes in joint tissue, and it can cause a variety of arthritis when abnormal (Mei et al., 2019; Alharbi et al., 2022). FLS are important factors in osteoremodeling in arthritis, and Wnt1/β-catenin signaling pathway exert marked effects in the survival of FLS cells (Dinesh et al., 2020). Studies have shown that abnormal Wnt1/β-catenin signaling pathway is the main mechanism of RA (Miao et al., 2013). In RA, Wnt1/β-catenin pathway signal transduction results in polymorphic changes of osteocytes/chondrocytes, causing bone erosion and cartilage degradation (Sujitha et al., 2020). Wnt1 is mainly expressed in synovial cells, after the activation of Wnt signaling pathway, the expression of β-catenin increases, which promotes the secretion of inflammatory factors. RA-FLS is activated and induces RA, Cai et al. (2022) confirmed that blocking Wnt1/β-catenin signaling pathway and inhibiting TNF-induced migration, invasion and inflammation of RA-FLS cells can effectively alleviate adjuvant arthritis (AA). Some miRNAs can be used as inhibitors of Wnt1/β-catenin signaling pathway to further prevent RA (Miao et al., 2014; Sujitha et al., 2020) showed that berberine could inhibit Wnt1/β-catenin signal transduction through miR-23a activation, thereby improving RA. Wnt1/β-catenin signaling pathway controls bone and joint development and is closely related to the pathogenesis and progression of OA (Ahmad et al., 2020). IL-1β-induced chondrocyte degeneration may be accompanied by activation of Wnt/β-catenin pathway, which exerts an important effect in the degeneration and destruction of OA articular cartilage (Su et al., 2012). Sry-box transcription factor 9 (SOX9) has the function of promoting cartilage anabolism, and abnormal expression may lead to OA (Carmon et al., 2023; Alahdal et al., 2021) showed that activation of Wnt1/β-catenin pathway inhibited the expression of SOX9 and collagen type II, and impaired the cartilage differentiation and regeneration of MSCs in OA patients. (Li et al., 2022) demonstrated that MiR-376c-3p from Adipose mesenchymal stem cell (ADSC) derived exosomes regulated the Wnt1/β-catenin signaling pathway by targeting WNT3 or WNT9a, improving chondrocyte degradation and synovial fibrosis induced by OA. Lietman et al. (2018) found that regulation of Wnt pathway could improve OA symptoms in surgery-induced mouse OA model. Mei et al. (2019) confirmed that inhibition of Wnt1/β-catenin pathway signal transduction and regulation of β-catenin stability in macrophages could effectively improve GA. Berberine can induce Dvl-1 inhibitor-CYLD to inhibit the expression of FZD4, LRP5 and Dvl-1, regulate the Wnt1/β-catenin signaling pathway in adjuvant arthritis FLS cells, and reduce the expression level of intracellular β-catenin, thus improving arthritis (Shen et al., 2020). Palmatine inhibits the progression of OA by regulating the Wnt1/β-catenin signaling pathway (Xuan et al., 2019). Therefore, the Wnt1/β-catenin signaling pathway can be used as a potential target for treating various types of arthritis (Zhou, 2014; Shang et al., 2021).

#### 3.2.2 PI3K/AKT/mTOR signaling pathway

PI3K/AKT/mTOR signaling pathway is mainly mediated by growth factor signal transduction to lipid metabolism, protein synthesis and cell proliferation and survival, and other physiological processes, and it is related to inflammation, autoimmune diseases and hematological malignancies, affecting cell proliferation, differentiation, metastasis and apoptosis (Foster et al., 2012; Abeyrathna and Su, 2015; Liu et al., 2021). The PI3K/AKT/mTOR signaling pathway is crucial for the normal metabolism of joint tissues and is closely related to the occurrence and development of OA and RA (Sun et al., 2020; Zhou et al., 2023; Zhang et al., 2001) found that the spontaneous and induced activation of AKT and the level of pAKT in RA patients were higher than those in OA patients. (Dinesh and Rasool, 2019). demonstrated that PI3K/AKT signaling pathway could be used as a key target for RA treatment, and inhibition of abnormal activation of PI3K/AKT signaling pathway played a key role in the prevention and treatment of RA (Ansari et al., 2022; Hashiramoto et al., 2007) confirmed that abnormal PI3K/AKT signaling pathway can lead to RA synovial overgrowth and joint destruction. Abnormal activation of PI3K/AKT signaling pathway can increase the expression level of anti-apoptotic genes in synovial cells of RA patients, and then leads to the exacerbation of RA disease (Harris et al., 2009; Smith et al., 2010). Wang (Wang, 2020) found that downregulation of PI3K/AKT pathway and inhibition of over-activation of AKT could effectively improve RA. Studies have shown that activation of PI3K/AKT signaling pathway leads to accelerated proliferation of FLS cells in AA and aggravation of the course of arthritis (Dinesh and Rasool, 2019). Abnormal activation of PI3K/AKT/mTOR signaling pathway will destroy the normal function of cartilage and subchondral bone (Sun et al., 2020). As immune cells, synovial macrophages are closely related to the occurrence and development of OA. Activated macrophages are regulated by the PI3K/AKT signaling pathway, and their activation status is highly correlated with the severity of OA (Zhang et al., 2020). Inhibition of PI3K/AKT/mTOR signaling pathway activates autophagy, promotes anabolism and inhibits catabolism of OA chondrocytes, and effectively treats OA (Wang, 2022). Studies have shown that quercetin regulates PI3K/AKT signaling pathway to improve arthritis by binding to and inhibiting PI3K in mouse epidermal cells to inhibit AKT phosphorylation (Khan et al., 2019). Berberine delays the progression of osteoporosis, RA and OA by regulating the PI3K/AKT signaling pathway (Wong et al., 2020). Therefore, the key proteins in PI3K/AKT signaling pathway can be used as potential targets of CC in the treatment of arthritis for in-depth study.

**FIGURE 3:**
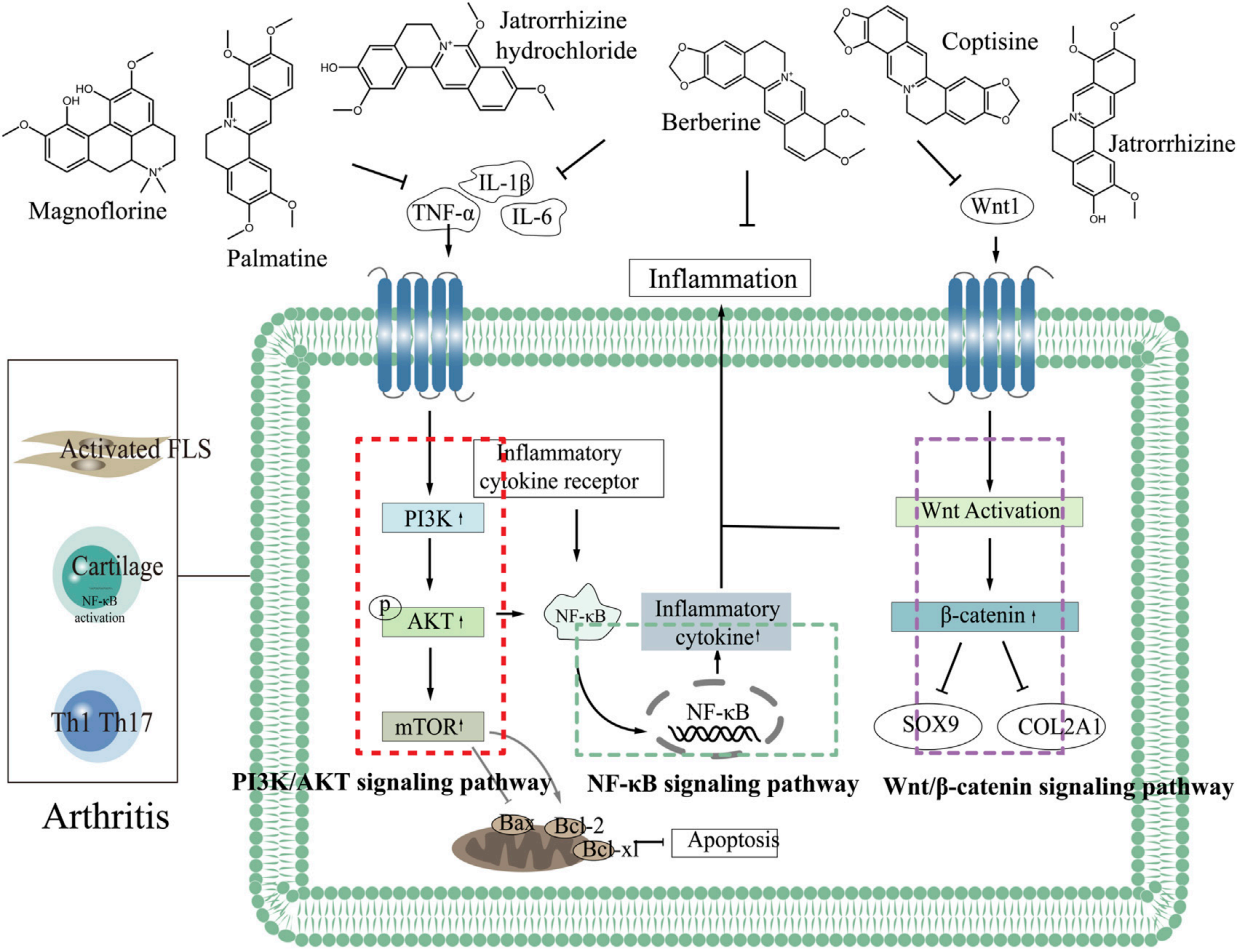
The chemical constituents and the pathways involved in the treatment of arthritis by CC.

By combining the pathogenesis of three types of arthritis with the related targets and pathways of CC in treating arthritis, we elucidated the mechanism, providing a new idea for the development of CC as a candidate drug for treating arthritis. Most of the components in CC in treating arthritis are alkaloids. Six components including berberine in CC have good effects in the treatment of arthritis, which can inhibit the expression of TNF-α, IL-6, IL-1β through PI3K/AKT, Wnt1/β-catenin and NF-κB signaling pathways, thereby reducing the inflammatory response and achieving the purpose of treating arthritis (Figure 3 demonstrated the chemical constituents and the pathways involved in the treatment of arthritis by CC).

## 4 Deep exploration based on potential therapeutic targets for arthritis

In this review, we have summarized the targets involved in the pathogenesis of arthritis, elaborated potential therapeutic targets, and provided a new idea for the exploration of the mechanism of CC in the treatment of arthritis. Studies have shown that CC can treat a variety of arthritis, and it is better for OA, RA and GA. CC has the characteristics of multiple components, multiple targets, and multiple pathways in the treatment of arthritis. Currently, IL-10, IL-1β, IL-6, MMP-3, TNF-α and other targets have been verified to play a role in treating arthritis by CC (Table 2) (Sun et al., 2011; Yuan et al., 2014; Kienhorst et al., 2015; Huang et al., 2019; Thomas, 2020; Che et al., 2021; Li et al., 2021; Wu et al., 2021; Arra et al., 2022; Zhang et al., 2022; Cao et al., 2022; Du et al., 2022; Pacifici, 2022; Wirth et al., 2022; Xiao et al., 2022; Yang et al., 2022; Sun et al., 2023a; Barbarroja et al., 2023; Li et al., 2023; Brix et al., 2023; Wang et al., 2023b; Wang et al., 2023; Ghosh et al., 2023; González-Chávez et al., 2023; Hecquet et al., 2023; Jahantigh et al., 2023; Ji et al., 2023; La Bella et al., 2023; Leung et al., 2023; Lin et al., 2023; Luo et al., 2023; Zeng et al., 2023; Zhang et al., 2023). However, the types of arthritis treated with these targets are not completely clear. Targets, β2-adrenergic receptor (ADRB2), AHR, CRP, IRF1, prostaglandin G/H synthase 1 (PTGS1), SPP1 and other targets, exert key effects in the pathogenesis of arthritis, but role of them in the treatment of arthritis with CC has not been confirmed (Table 3) (Xu et al., 2004; Koskela et al., 2012; Ko et al., 2013; Li et al., 2013; Xue et al., 2014; Song et al., 2016; Trzybulska et al., 2018; Bonelli et al., 2019; Chu et al., 2019; Lin et al., 2019; Sun et al., 2020; Orecchini et al., 2020; Yang et al., 2020; Nalesso et al., 2021; Tang et al., 2021; Xu and Xu, 2021; Zou et al., 2021; Balasundaram et al., 2022; Cai et al., 2022; Liu et al., 2022; Wang et al., 2022; Zhang et al., 2022; Zhang et al., 2022; Han et al., 2022; Man et al., 2022; Nguyen et al., 2022; Qian et al., 2022; Tavallaee et al., 2022; Zhuang et al., 2022; Afnan et al., 2023; Baggio et al., 2023; Sun et al., 2023b; Zhou et al., 2023; Fan et al., 2023; Huang et al., 2023; Ning et al., 2023; Qin et al., 2023; Skougaard et al., 2023; Tu et al., 2023; Xie et al., 2023; Yan et al., 2023; Yu et al., 2023; Zhao et al., 2023). We have elaborated on the role of targets such as MMP-3, IL-1β, MAPK, IL-6, ADRB2, AHR, CRP, CAV1, CXCL2, SPP1 and other targets in treating arthritis, explored the potential targets and mechanisms of CC in treating arthritis, analyzed the feasibility of CC as an anti-arthritis drug, and provided a theoretical basis for subsequent research.

**TABLE 2:** Verified targets involved in the treatment of arthritis by CC.

Gene	Protein name	Arthritis type	Expression	Literature
CCL2	C-C motif chemokine 2	OA, RA	Upregulated	Wang et al. (2023b), Luo et al. (2023)
IL-10*	Interleukin-10	RA, OA, PsA, CIA, GA, Juvenile idiopathic arthritis (JIA), AIA	Downregulated	Barbarroja et al. (2023), Ghosh et al. (2023), González-Chávez et al. (2023), Jahantigh et al. (2023), Ji et al. (2023), La Bella et al. (2023), Zeng et al. (2023), Zhang et al. (2023)
IL-1β*	Interleukin-1 beta	RA, OA, CIA, GA	Upregulated	Yuan et al. (2014), Huang et al. (2019), Arra et al. (2022), Yang et al. (2022)
IL-6*	Interleukin-6			Yuan et al. (2014), Kienhorst et al. (2015), Thomas, 2020; Pacifici (2022)
AKT1	RAC-alpha serine/threonine-protein kinase	RA, OA, CIA	Upregulated	Che et al. (2021), Xiao et al. (2022), Li et al. (2023b)
CXCL8*	Interleukin-8	RA, GA	Upregulated	Kienhorst et al. (2015), Du et al. (2022)
TNF-α*	Tumor necrosis factor	RA, OA, GA, PsA	Upregulated	Sun et al. (2023a), Wang et al. (2023c), Hecquet et al. (2023), Leung et al. (2023)
MAPK1	Mitogen-activated protein kinase 1	RA, OA, CIA	Downregulated	Li et al. (2021), Zhang et al. (2022b), Cao et al. (2022)
MMP-3*	Matrix metallopeptidase-3	RA, OA, JIA, AIA, PsA, CIA	Upregulated	Sun et al. (2011), Huang et al. (2019), Wu et al. (2021), Wirth et al. (2022), Brix et al. (2023), Lin et al. (2023)

**TABLE 3:** Targets in the pathogenesis of arthritis.

Target	Protein ID	Arthritis type	Expression in arthritis	Literature
ADRB2	Beta-2 adrenergic receptor	OA, RA	Upregulated	Xu et al. (2004), Sun et al. (2020b)
AHR^a^	Aryl hydrocarbon receptor	RA, CIA, OA	Downregulated	Zhuang et al. (2022), Huang et al. (2023)
Bax	Apoptosis regulator BAX	OA, RA, CIA, psoriatic arthritis (PsA), AIA	Downregulated	Shen et al. (2022), Zhou et al. (2023a), Baggio et al. (2023)
Bcl-2	Apoptosis regulator Bcl-2		Upregulated	
BCL2L1	Bcl-2-like protein 1	OA, RA	Downregulated	Koskela et al. (2012), Yang et al. (2020)
BIRC5	Baculoviral IAP repeat-containing protein 5	RA	Upregulated	Balasundaram et al. (2022)
CAV1^a^	Caveolin-1	CIA, RA, AA	Upregulated	Song et al. (2016), Trzybulska et al. (2018), Zou et al. (2021)
CCND1	G1/S-specific cyclin-D1	OA	Upregulated	Man et al. (2022)
COL3A1	Collagen alpha-1(III) chain	OA	Upregulated	Han et al. (2022)
CRP^a^	C-reactive protein	RA, AIA, PsA	Upregulated	Afnan et al. (2023), Skougaard et al. (2023), Yu et al. (2023)
CDKN1A	Cyclin-dependent kinase inhibitor 1	RA, OA, CIA	Downregulated	Chu et al. (2019), Liu et al. (2022b), Fan et al. (2023)
CXCL2^a^	C-X-C motif chemokine 2	RA, CIA, PsA	Upregulated	Zhang et al. (2022c), Jie et al. (2022), Nguyen et al. (2022)
HMOX1	Heme oxygenase 1	OA	Downregulated	Nalesso et al. (2021)
IRF1^a^	Interferon regulatory factor-1	RA, OA	Upregulated	Bonelli et al. (2019), Zhao et al. (2023)
MMP-2	Matrix metalloproteinase-2	RA, OA, CIA, AIA	Upregulated	Ko et al. (2013), Li et al. (2013), Xue et al. (2014), Cai et al. (2022a)
MMP-9	Matrix metalloproteinase-9			
NOS2	Nitric oxide synthase	OA, GA, CIA	Upregulated	Orecchini et al. (2020), Zhang et al. (2022d), Zhou et al. (2023b)
PPARG	Peroxisome proliferator-activated receptor gamma	RA, OA	Downregulated	Tavallaee et al. (2022), Qin et al. (2023)
PTGS1	Prostaglandin G/H synthase 1	RA, OA	Upregulated	Qian et al. (2022), Tu et al. (2023)
PTGS2	Prostaglandin G/H synthase 2			
SPP1^a^	Secreted phosphoprotein 1	RA, OA, CIA	Upregulated	Lin et al. (2019), Cai et al. (2022b), Du et al. (2022)
STAT1	Signal transducer and activator of transcription 1-alpha/beta	RA, OA, CIA	Upregulated	Tang et al. (2021), Xu and Xu (2021), Sun et al. (2023b)
TGFB1	Transforming growth factor beta-1 proprotein	RA, OA, GA, CIA	Upregulated	Wang et al. (2022b), Ning et al. (2023), Xie et al. (2023), Yan et al. (2023)

^a^ Key targets in the pathogenesis of arthritis.

IL-6 plays an important role in the development of RA (Cheng et al., 2022), and it is associated with inflammatory response and cartilage loss in the pathogenesis of OA (Hou et al., 2020). IL-10 is an important anti-inflammatory and immunosuppressive cytokine that not only prevents the occurrence of arthritis, but also has an inhibitory effect on the development of arthritis (Charbonnier et al., 2010). IL-1β is related to the inflammation of synovium, which can affect the normal metabolism of chondrocytes, change the structure and function of osteocytes, promote the apoptosis of chondrocytes and the decomposition of cartilage matrix, and it plays a key role in the pathogenesis of arthritis (Bai, 2021). TNF-α is a pro-inflammatory cytokine secreted by membrane-forming FLS and mainly distributed in the joint space of RA, anti-TNF therapy is the preferred therapy for severe RA patients (Taylor et al., 2022). Activated ADRB2 in osteoblasts stimulates osteoclastogenesis and upregulates RANKL expression, thereby reducing bone formation and promoting bone resorption, leading to bone loss and osteoarthritis (Ma et al., 2011; Liang et al., 2018). AHR can be used as a key target for the treatment of RA (Xi et al., 2022), it has a variety of potential roles in the immune system. Various natural products can alleviate synovial inflammation and restore immune balance in RA patients by binding to AHR in fibroblast-like synovial cells and T cells (Stockinger et al., 2014; Hui and Dai, 2020). The expression of anti-apoptotic proteins Bcl-xl increased significantly in arthritis patients (Chen et al., 2016). CAV1 is a regulator of various cell signaling pathways. Reducing the expression of CAV1 can inhibit the expression of IL-1β-induced CCL2 mRNA and promote the apoptosis of RA-FLS (Li et al., 2017). CXCL2 promotes osteoclast formation and is associated with bone erosion in RA. Studies have shown that blocking expression of CXCL2 may be a means of treating RA (Wang et al., 2021). IRF1 promotes chondrogenesis of hADSCs by up-regulating HILPDA level, and it provides a new biomarker for the treatment of osteoarthritis (Zhao et al., 2023). As a proteolytic enzyme secreted by synovial fibroblasts, MMPs are involved in the pathogenesis of arthritis and play an important role in inflammatory response and joint destruction (Murphy and Nagase, 2008). The levels of MMP-2 and MMP-9 are elevated in the serum of RA patients, which can reflect the early inflammatory level of RA (Hu et al., 2011). MMP-3 plays a key role in the pathogenesis of RA and is one of the key indicators for the treatment of RA (Lerner et al., 2018) Studies have shown that when PTGS1 is overexpressed, the migration and invasion of OA synovial cells increase, and the apoptosis rate decreases (Wang et al., 2019). In collagen induced arthritis, SPP1 secreted by FLSs promotes the formation of osteoclast through PI3K/AKT signals. Regulating the expression of SPP1 gene in FLSs may be a potential method to treat RA bone injury in the joint microenvironment (Cai et al., 2022). Therefore, ADRB2, AHR, CRP, IRF1, PTGS1, SPP1 and other targets can be used as potential targets for CC in the treatment of arthritis, and it is of great significance to explore its role of CC in the treatment of arthritis.

## 5 Conclusion

TCM plays an important role in the treatment of arthritis due to its multi-component, multi-efficacy and multi-target characteristics. CC, as an antibacterial and anti-inflammatory Chinese medicine, has a good effect in treating arthritis. In this review, we have summarized the chemical constituents, targets and related pathways of CC in treating arthritis, discussed the mechanism of CC in the treatment of arthritis from the molecular level, clarified the potential targets, and provided reasonable directions for clinical treatment of arthritis. Berberine, palmatine, coptisine, jatrorrhizine, magnoflorine and jatrorrhizine hydrochloride in CC have the effect of treating arthritis, especially berberine can treat a variety of arthritis, such as RA, OA and GA. Berberine improves arthritis by reducing cell inflammation, improving chondrocyte function, promoting cartilage synthesis and repair, and promoting uric acid excretion. Palmatine can significantly block the Wnt1/β-catenin signaling pathway, protect chondrocytes and knee cartilage, and inhibit the progression of OA. At the molecular level, six components including berberine can improve RA, OA, GA and other types of arthritis by regulating PI3K/AKT, Wnt1/β-catenin and NF-кB signaling pathways. IL-10, IL-1β, IL-6, MMP-3, TNF-α, COX-2, TGF-β, Caspase-1, MAPK and other targets have been confirmed to play key roles in the treatment of arthritis by CC, and can be used as targets for clinical treatment of arthritis, providing scientific basis for the development of rational targeted drugs for the treatment of arthritis. AHR, CAV1, CRP, CXCL2, IRF1, SPP1 and other targets play important roles in the pathogenesis of arthritis and can be used as key targets for the treatment of arthritis. However, the role of them of CC in the treatment of arthritis remains to be further verified.

All in all, berberine, palmatine, coptisine, jatrorrhizine, magnoflorine and jatrorrhizine hydrochloride in CC can effectively treat arthritis, and have been proved. At the molecular level, CC plays a critical role in the treatment of arthritis by regulating NF-κB, Wnt1/β-catenin and PI3K/AKT/mTOR signaling pathway, inhibiting the expression of IL-6, IL-1β, MMP-3 and TNF-α. In this review, we have concluded with a summary and our insights on the chemical components, targets and pathways of CC in the treatment of arthritis, and discussed the relevant mechanism and potential targets, providing scientific basis for CC in the clinical treatment of arthritis.

## Figures and Tables

**Table:** 

**CC**	Coptis chinensis
**RA**	Rheumatoid arthritis
**OA**	Osteoarthritis
**GA**	Gouty arthritis
**IL-1β**	Interleukin-1β
**IL-6**	Interleukin-6
**IL-1**	Interleukin-1
**TNF-α**	Tumor necrosis factor-α
**iNOS**	Inducible nitric oxide synthase
**IL-10**	Interleukin-10
**IL-17**	Interleukin-17
**MAPK**	Mitogen-activated protein kinase
**MMP-1**	Matrix metalloproteinase-1
**MMP-2**	Matrix metalloproteinase-2
**MMP-3**	Matrix metalloproteinase-3
**MMP-9**	Matrix metalloproteinase-9
**MMP-13**	Matrix metalloproteinase-13
**MMPs**	Matrix metalloproteinases
**AHR**	Aryl hydrocarbon receptor
**CAV1**	Caveolin-1
**CRP**	C-reactive protein
**CXCL2**	C-X-C motif chemokine 2
**IRF1**	Interferon regulatory factor-1
**SPP1**	Secreted phosphoprotein 1
**TCM**	Traditional Chinese medicine
**COX-2**	Cycloooxygenase-2
**MSU**	Monosodium urate
**UA**	Uric acid
**FLS**	Fibroblast-like synoviocytes
**Bax**	Apoptosis regulator BAX
**Bcl-2**	Apoptosis regulator Bcl-2
**TGF-β**	Transforming growth factor-β
**IFN-γ**	Interferon-γ
**RORγt**	RAR-related orphan receptor γt
**CCN2**	Connective tissue growth factor
**HIF-1α**	Hypoxia inducible factor 1α
**PGE2**	Prostaglandin E2
**NLRP3**	NOD-like receptor thermal protein domain 3
**AGA**	Acute gouty arthritis
**NALP3**	Nucleotide-binding oligomerization domain-like receptor 3
**CXCL12**	C-X-C motif chemokine 12
**CXCR4**	C-X-C chemokine receptor 4
**AA**	Adjuvant arthritis
**ADSC**	Adipose mesenchymal stem cell
**ADRB2**	β2-adrenergic receptor
**PTGS1**	Prostaglandin G/H synthase 1
**SOX9**	Sry-box transcription factor 9
